# Medical and Physical Intelligence

**Published:** 1805-10-01

**Authors:** 


### MEDICAL AND PHYSICAL INTELLIGENCE.

[ FOREIGN AND DOMESTIC. ]

The Collegium Medicum et Sanitatis, at Berlin, by order of His Majesty the King of Prussia, proposes a prize of two hundred ducats for the best answer, and a prize of one hundred ducats for the next best answer to the following questions. The College invites all professional men, who had an opportunity of treating the yellow fever, to communicate their observations, and to give their opinions on the following points:

1. It being proved by experience, that the yellow fever is propagated by contagious matter, is it beyond doubt, that this matter adheres to lifeless substances, so far as to infect by touch, any sound and healthy person, and so to carry to a distance, the yellow fever?

2. Taking the possibility of such an infection for granted, by what experiments, facts, or observations, can this opinion be made probable, or brought to a certainty?

3. Is it probable, or proved, that the contagious matter of the yellow fever, is a produce of this disease, or contained particularly in one or more animal secretions? and in which of them?

4. Are the chemical properties of this matter already so known, as to enable us to administer chemical antidotes, by which the contagious matter may be destroyed or decomposed? Or are there any preventives, and in what manner are these to be exhibited?

5. How long does the contagious matter keep the power to infect, and how long are the substances, impregnated with this matter, able to spread the infection?

6. Is there any difference among lifeless substances, to assume easier and sooner the contagion, and to keep it longer? Are there any who do not receive the infection at all?

7. Has the disease of North America, the South of Spain, and Leghorn, been of the same nature? or has any material difference existed?

8. Is the yellow fever an endemical disease of the sea-shore, or is it to be feared, that it may have the same deleterious effect at a great distance from the sea, or on the Continent?

The Answers must be either in Latin, German, or French, directed to the Royal Collegium Medicum et Sanitatis, at Berlin, before January 1, 1807, without the name of the author, in the usual manner.

---

His

His Majesty the King of Prussia has given orders, that as the mineral fumigations of Guyton Morveau, are proved to be the safest preventive against the yellow fever, they shall be adopted in all Prussian harbours, and in all vessels under quarantine, or coming from suspected places.

---

A German gentleman, travelling through different places in Spain, at the time when the yellow fever made its ravages, observed, that of all kind of birds, the sparrows only had some notion of the dangerous influence of this disease, so far that they left the houses when the infection had taken place, and by no allurement were to be induced to return, while other birds fell victims to their ignorance. The inhabitants therefore considered the continuance of the sparrows in a dwelling house, as a certain proof of its being free from the contagion.

---

By a ten year's comparison of the Bills of Mortality of Vienna, the number of deaths upon an average, yearly amounted to 14600, and among these, 835 children fell victims to the natural small-pox every year. But since the introduction of the cow-pox, no more than 164 children died of the small-pox in the year 1801; in the year 1802, only sixty-one; in the year 1803, but thirty-seven; and in the year 1804, only two children, and of these, one belonged to foreign travelling parents.

---

The Societé de Medicine at Brusselles, has proposed a Prize for the year xiii. of the value of two hundred francs, for the following question:

Has the night any influence on sick persons?

Are there any diseases where this influence is more or less visible?

What is the physical cause of this influence?

Letters to be directed to Dr. Fournier, secretary to the society, either in French or Latin.

---

*Preparation of the true Copal Varnish.*—Take two parts of gum-copal, reduced to a fine powder, and washed repeatedly in water to free it from the woody fibres; introduce it into a flask, and pour over it four parts of pure oil of rosemary; digest the mixture in a gentle heat for three days, or longer, after which add as much highly rectified spirit of wine as is deemed necessary, and suffer it to remain undisturbed uutil the impurities subside; then decant the varnish.

---

M. Proust says, that the sulphate of copper and the nitrate, with a minimum of acid, verdigris, the native and artificial muriates, cendre blue, the carbonate, &c. all yield to potash both their acids and hydrites. Potash, tinged with hydrate of copper, throws down the hydrate on being mixed with water, and all the oxydooxydo-alkaline solutions follow the same law. Slaked lime, shaken in a bottle with carbonate of copper and water, produces a fine cendre blue in about twelve hours; after which, as lime deprives potash of its carbonic acid entirely, and potash is one of the strongest attractors of acids known, it is impossible that it should not have the same power over an oxyde, and that oxyde possessed of the weakest attraction of any.

---

M. Dobeimer proposes the following method to make white lead. Dissolve litharge in weak nitric acid, and precipitate this solution with prepared chalk. The precipitate washed and dried affords a ceruse of the whiteness of snow.

---

Dr. Buchholz has improved the preparation of the naphtha aceti, in the following manner. Upon sixteen ounces of well dried and powdered acetated lead, a mixture of six ounces of concentrated acid of brimstone, and nine ounces of spirit of wine, is to be poured, and by a mild fire the quantity of nine or ten ounces liquid to be drawn over. If this liquid be mixed afterwards with the third part of lime-water, nearly six ounces of æther aceti will be gained. When proper care has been taken, that the liquid which is drawn over, does not contain any acetated lead, the æther may be used without any further preparation. Should this not be the case, the æther must again be rectified.

---

The Lectures at the London Hospital will begin on Oct. 1.

Theory and Practice of Physic, by Dr. Cooke.

Chemistry, by Dr. Hamilton and Dr. Yelloly.

Theory and Practice of Midwifery, by Dr. Dennison.

Anatomy, Physiology, and the Operations of Surgery, by Mr. Headington and Mr. Frampton.

Anatomical Demonstrations and Dissections, by Mr. Armiger.

Surgery, by Mr. Headington.

Clinical Observations on Cases under Treatment, by Sir Wm. Blizard and Mr. Thomas Blizard.

Application to be made to Mr. Price, Apothecary, at the Hospital.

---

Mr. Home's Lectures on the principal Operations in Surgery, given gratuitously to the pupils of St. George's Hospital, will commence on Thursday, the 24th of October next.

Mr. Gunning, in the course of the season, will likewise give six lectures on Syphilitic Complaints.

---

Dr. Hooper will commence a Course of Lectures on the Theory and Practice of Physic, Materia Medica, and Pharmaceutical Chemistry, on Wednesday, Oct. 16, at his Museum, Cork Street, Burlington Gardens. Particulars may be known by applying at his house, No. 13, Holles Street, Cavendish Square.

---

Mr.

Mr. Moor, Surgeon-Dentist to Her Royal Highness the Dutchess of York, will commence a Course of Lectures on the Structure and Diseases of the Teeth, on November 5, in which will be explained the complete Practice of the Dentist. Further particulars may be known at his house, No. 6, Palsgrave Place, Temple.

---

Dr. Reid, of the Finsbury Dispensary, will commence his Course of Lectures on the Theory and Practice of Medicine, on Thursday, October 17, at eight o'clock in the evening, at No. 7, Cateaton Street, (late Paul's Head Tavern) at which place the subsequent lectures will be delivered, at twelve o'clock, every Tuesday, Thursday, and Saturday, until the middle of January.

---

Mr. Thomas will commence his Course of Lectures on the Principles and Operations of Surgery, on Monday, Oct. 7, at his house in Leicester Place, where a prospectus may be had, or at the Anatomical Theatre, Windmill Street.

---

Mr. Ring has sent to the press An Answer to Dr. Moseley, containing a Defence of Vaccination, which will be ready for publication in a few days.

---

Mr. Thomas Luxmore, Surgeon Extraordinary to His Royal Highness the Prince of Wales, Surgeon to the Honourable Artillery Company and to the Eastern Dispensary, will in a few days publish a Manual of Anatomy and Physiology, reduced as much as possible to a tabular form, for the purpose of facilitating to students the acquisition of these sciences.

---

On Wednesday, September 8, came on the election of Surgeon to the Finsbury Dispensary, occasioned by the resignation of Mr. Ricards. On casting up the ballot, there appeared for

| | |
|---|---|
| Mr. John Taunton – – – | 350 |
| Mr. Samuel Smith – – – | 143 |
| | 207 Majority. |

On which Mr. Taunton was declared duly elected Surgeon to the above charity.

